# A gaping research gap regarding the climate change impact on health in poor countries

**DOI:** 10.1007/s10654-017-0258-7

**Published:** 2017-06-01

**Authors:** Rainer Sauerborn

**Affiliations:** Heidelberg University, Heidelberg, Germany

Compared to other disciplines and sectors, the epidemiological and medical communities have been slow to turn their interest to the climate impacts on health [1, 2]. Embarrassingly, there are particularly few studies in those populations, where the exposure to health damaging climate change is highest, while adaptation capacity is lowest.

Why is this so?

The first argument, which is often put forward, is the lack of valid and reliable health data in these countries, most of which are low or middle income. We hear this argument frequently from climate scientists, as well. Fortunately, this problem can be solved: high quality, long-term (up to 60 years), retrospective, mortality data sets do exist, comprising more than four million observed person-years [3, 4].

The second set of arguments is of more methodological nature. (1) our classical epidemiological tools, comparing exposed and control groups with respect to a risk factor are often not applicable, given the pervasiveness of climate change and the lack of control groups. (2) Climate change comprises different pathways, all of which have quite different health impacts: temperature, precipitation, ocean changes (pH, T, level) and air pollution.^1^ Moreover, gradual changes have different impacts from extreme events. (3) There is no such a thing as a new, or even typical “climate disease”. Rather, climate adds an incremental burden to some 80 already existing climate-sensitive diseases, from infectious to non-communicable diseases, undernutrition and injuries. The challenge is therefore to aggregate both mortality and morbidity impacts of a single disease under study, or ideally of all climate sensitive-diseases into a comprehensive, population-based metric for health outcomes, such as DALYs or QALYs. (4) The new risk factor “climate change” increases roughly exponentially over decades. Hence, no prospective observational study can be helpful in the time horizon that funders are willing to contemplate, i.e. 3–5 years. We have to learn from the past (see first argument). (5) Health impacts are likely to be influenced not only by a myriad of confounders and effect modifiers- social, economic, environmental and, of course, other risk factors for the disease under study- but also from adaptation efforts, both at the individual and societal levels. And finally (6), epidemiologists need to enter into deep cooperation with less familiar disciplines, probably more so than in any other fields of research: meteorology, climate science, agriculture, remote sensing and more.

What are some pathways to solve these problems?

One is about human brainpower: We need to interest and “enroll” the sharpest scientific minds into this scientific challenge. Unfortunately, we do a bad job at teaching the topic in a systematic way at our universities, West, East, North and South: my last count was 5 accredited formal short courses of 1–3-weeks durations worldwide on “climate change and health”. Massive Open Online Courses (MMOCs) can quickly disseminate knowledge and convey skills. However, the current four MOOCS on the topic are introductory or policy-focused and do not provide research skills. This, however, is the focus of a new MOOC on “Research Methods for Studying Climate Change Health”. This course is currently being developed by scientists of various disciplines from 7 universities: Harvard, Heidelberg, Charité Medical School (Berlin), the London School of Hygiene, Nottingham, Paris Descartes/Sorbonne-Cité, and Umeå. Theses universities are joined by the INDEPTH-network.org, as well as the Potsdam Institute for Climate Change Impact Research (PIK). The course is targeted to doctoral students and postdocs from everywhere wishing to study the impact of climate change on a disorder. The course will provide access to the type of long-term data-sets in (sub-)tropical countries, which I described in the second para above. In addition, participants will learn the required (to be adapted) tools in quantitative health sciences as well as an understanding of key tools from other disciplines (e.g. on using climate models, weather data, agricultural data, remotely sensed data etc.).

There are other road blocks: particularly our young colleagues are often put off by the lack of project funding in the area of climate change and health. Research funding organizations need to raise the number and volume of their calls in this arena. Often, the topic falls through the disciplinary “silos” of funding institutions: environment, health. Importantly, funders should not evaluate proposals as much on the PI’s past track records in this field, as this is a new research frontier and even the senior scientists in this field—globally maybe only two dozen—do not and cannot have a long publication track records in this field.

Journals, in turn, must accept that innovative research has higher risks, that interdisciplinary research is often penalized in reviews, due to “serial” evaluations by specialized scientists.

Finally, academic institution need to step up to the task and create careers, from junior research groups to senior positions. As an example: in Germany, there is currently not a single chair for “Climate change and health”.

## Footnotes

1 Short-lived climate active pollutants [5].
